# Integrated single-cell and spatial transcriptomic analyses identify TRIP6 as a prognostic and EMT-associated biomarker in colorectal cancer

**DOI:** 10.3389/fcell.2026.1874138

**Published:** 2026-07-22

**Authors:** Qiwei Cui, Xiaohong Gao, Xiaoqing Wu, Dongwei Zhang

**Affiliations:** 1 School of First Clinical Medicine, The First Affiliated Hospital of Anhui University of Chinese Medicine, Hefei, China; 2 Graduate School, Tianjin University of Traditional Chinese Medicine, Tianjin, China; 3 Department of Acupuncture and Tuina, Anhui University of Chinese Medicine Guoyitang Clinic, Hefei, China; 4 The First Department of Oncology, The First Affiliated Hospital of Anhui University of Chinese Medicine, Hefei, China

**Keywords:** colorectal cancer, drug repurposing, epithelial–mesenchymal transition, spatial transcriptomic, TRIP6

## Abstract

**Background:**

Colorectal cancer (CRC) is characterized by profound molecular heterogeneity and complex tumor–microenvironment interactions, which contribute to invasion, metastasis, and variable clinical outcomes. More effective prognostic biomarkers and therapeutic targets are still needed.

**Methods:**

We integrated single-cell RNA sequencing, spatial transcriptomics, multi-cohort bulk transcriptomic data, drug response prediction, and *in vitro* experiments to characterize malignant epithelial states in CRC and identify clinically relevant biomarkers and candidate therapeutics. Stemness, copy number variation, pathway activity, and cell–cell communication were analyzed at single-cell resolution. A prognostic model was established using epithelial marker genes and validated across TCGA and five GEO cohorts through a consensus machine-learning framework. Drug sensitivity was evaluated using CTRP and PRISM datasets, and candidate compounds were further prioritized through a network-based drug repositioning strategy. Spatial transcriptomics was used to define the tissue localization of key genes, and functional assays were performed to assess the role of TRIP6 in CRC cells.

**Results:**

Malignant epithelial cells exhibited increased stemness, frequent aneuploidy, enhanced glycolysis, along with upregulation of the Wnt/β-catenin and PI3K-AKT-mTOR signaling cascades. Cell–cell communication analysis revealed prominent extracellular matrix-related interactions linking cancer-associated fibroblasts and epithelial cells, suggesting a microenvironmental contribution driving epithelial–mesenchymal transition (EMT). The prognostic model showed stable predictive performance across independent cohorts. Among the model genes, TRIP6 was identified as a risk-associated candidate and was enriched at the tumor–stroma invasive boundary by spatial transcriptomic analysis. *In vitro*, TRIP6 knockdown suppressed CRC cell proliferation and migration, promoted apoptosis, and partially reversed EMT-associated marker expression, supporting a potential role for TRIP6 in malignant progression. In addition, drug prediction analyses identified several compounds with potential therapeutic relevance for high-risk patients.

**Conclusion:**

This study provides a multi-omic framework for understanding CRC progression and suggests that TRIP6 may be involved in linking microenvironmental signaling to invasive phenotypes, with potential value for prognostic stratification and therapeutic exploration.

## Introduction

Colorectal cancer (CRC) remains one of the most frequently diagnosed and lethal malignancies worldwide (Sung et al., 2021). In spite of notable progress in surgery, targeted therapy, and immunotherapy, outcomes for patients with advanced CRC are still poor because of its strong invasive and metastatic potential (Li Q. et al., 2024; Singh et al., 2024; Zhang et al., 2025; Yuan et al., 2024; Li Z. et al., 2024). Although the TNM staging system continues to guide routine clinical decision-making, it does not adequately capture the profound molecular heterogeneity within tumors (Li et al., 2023; Wu et al., 2026c; Wu et al., 2025b). As a result, patients with the same clinicopathological stage often exhibit substantially different disease courses and treatment responses. This underscores the need to further dissect the molecular mechanisms underlying CRC progression and to identify more precise prognostic biomarkers and actionable therapeutic targets.

The lethality of CRC is largely attributable to its capacity for invasion and metastasis, with epithelial–mesenchymal transition (EMT) serving as a central process that endows cancer cells with these aggressive traits (Lu et al., 2023). Importantly, EMT is not an isolated, tumor cell-autonomous event, but rather a dynamic program shaped by continuous interactions with the tumor microenvironment (TME) (Deng et al., 2023). The TME is a highly complex ecosystem in which malignant cells undergo profound stemness-associated dedifferentiation, widespread copy number variation (CNV), and metabolic rewiring, including the Warburg effect (Zhang et al., 2021; Wu et al., 2026b). Together, these intrinsic alterations confer remarkable cellular plasticity on malignant epithelial cells and create a permissive context for EMT activation. Of particular interest, stromal components—especially cancer-associated fibroblasts (CAFs)—engage in extensive paracrine crosstalk with malignant epithelial cells through extracellular matrix (ECM) signaling (Zhou et al., 2024). Such receptor–ligand interactions are widely regarded as major candidate regulators that initiate and sustain EMT, thereby facilitating local invasion and distant dissemination in CRC. However, although the importance of EMT in tumor metastasis is well recognized, how distinct microenvironmental cell populations cooperate to trigger EMT within complex three-dimensional tissue architecture, particularly at the invasive front, remains insufficiently understood at single-cell and spatial resolution (Lu et al., 2025; Pan et al., 2021; Willier et al., 2011; Wu et al., 2025a). Likewise, the key molecular determinants governing this malignant transition have yet to be systematically defined.

To address these questions, we integrated single-cell RNA sequencing, spatial transcriptomics, multi-cohort bulk transcriptomic data, and *in vitro* experiments. Using single-cell analysis, we systematically characterized stemness features, CNV evolution, and metabolic reprogramming in the CRC microenvironment, and delineated the extensive communication network between CAFs and epithelial cells. We then applied a consensus framework comprising 36 distinct combinations of machine-learning methods to construct and validate a robust prognostic model across large independent cohorts. Notably, TRIP6 was recognized as a candidate factor associated with poor prognosis, and spatial transcriptomic analysis further showed that TRIP6 was preferentially enriched at the tumor–stroma invasive boundary. Finally, a series of cell-based experiments showed that TRIP6 knockdown markedly inhibited CRC cell proliferation and migration, promoted apoptosis, and reversed the malignant EMT phenotype. Collectively, our study provides a multi-omic spatiotemporal view of CRC progression and identifies a promising molecular target for prognostic stratification and therapeutic intervention.

## Methods

### Data collection and processing

Single-cell RNA sequencing datasets (GSE161277 and GSE132465) were obtained from the GEO database. The combined dataset consisted of 40 samples (27 colorectal cancer and 13 normal colon samples). Downstream data processing was strictly performed using the R package Seurat (v4.4.1). To ensure high data quality, raw expression matrices underwent a step-by-step filtering process. Initial quality control removed cells with fewer than 250 detected genes or a mitochondrial gene percentage greater than 10%. To identify true single cells, ambient RNA contamination was corrected using decontX, and doublets were removed by DoubletFinder. The final filtering criteria retained high-quality cells with more than 500 expressed genes, less than 20% mitochondrial transcripts, and less than 1% red blood cell genes. Batch effects across different datasets were corrected using the Harmony algorithm. Cell types were annotated based on Reference (Wang et al., 2023) and the CellMarker 2.0 (Hu et al., 2023) database. Furthermore, cellular stemness was analyzed using CytoTRACE, and copy number variations at the single-cell level were calculated via CopyKAT.

Bulk transcriptomic data and matched clinical information for colon adenocarcinoma (COAD) were retrieved from TCGA, while five external GEO cohorts (GSE175383 (Smith et al., 2010), GSE331134 (de Sousa et al., 2011), GSE395825 (Marisa et al., 2013), GSE143336 (Jorissen et al., 2009), and GSE388327 (Tripathi et al., 2014)) served as validation datasets. Samples lacking complete survival information were excluded from prognostic analyses. Differential expression between tumor and normal tissues was assessed using limma, with |log2FC| > 0.8 and *P* < 0.05 as the cutoff.

### Functional enrichment and pathway activity scoring

Differentially expressed genes were functionally annotated using clusterProfiler (Yu et al., 2012) for GO (BP, CC, and MF) and KEGG (Kanehisa and Goto, 2000) enrichment analyses. Significant enrichment was defined as an FDR-adjusted *P* < 0.05. Furthermore, to quantify specific functional states at the cellular level, the AUCell algorithm was executed. Activity scores for four core biological modules (immunity, metabolism, signaling, and proliferation) were computationally derived to assess the continuous activation levels of these target pathways.

### Cellchat analysis

Intercellular communication networks were computationally inferred using the R package CellChat (v2.0) (Jin et al., 2021). Based on the annotated single-cell subpopulations, ligand-receptor interaction probabilities were mapped against the curated CellChatDB.human database. The algorithm systematically quantified the interaction strength and dynamic crosstalk across 32 distinct signaling pathways to decode the cellular microenvironment.

### Construction and optimization of the machine-learning prognostic model

Epithelial marker genes were first analyzed by univariate Cox regression to identify prognosis-related candidates. To develop a reliable consensus signature, we implemented a machine-learning strategy incorporating 36 algorithm combinations. The candidate methods included Random Survival Forest (RSF), Elastic Net (Enet), Lasso, Ridge, Stepwise Cox, CoxBoost, Partial Least Squares Regression for Cox (plsRcox), Supervised Principal Component (SuperPC), Generalized Boosted Regression Modeling (GBM), and survival support vector machine (survival-SVM). Model construction proceeded as follows: a prognostic feature matrix was established, all 36 algorithm combinations were trained in the TCGA-COAD cohort under a leave-one-out cross-validation (LOOCV) scheme, and model performance was subsequently tested in external validation datasets. Harrell’s concordance index (C-index) was calculated for each model across all cohorts, and the combination with the best mean C-index was selected as the final model. Patients were then divided into high- and low-risk groups according to the median risk score, and survival differences were assessed using Kaplan–Meier analysis.

### Spatial transcriptomics data processing and topographical mapping

Spatial transcriptomic (ST) matrices for colorectal cancer (ST-colon1/4) (Wu et al., 2022) were acquired from public repositories. Analytical pipelines were strictly executed in R based on the standard Seurat spatial framework. Raw spatial counts were normalized utilizing the SCTransform algorithm. Dimensional reduction was computed via Principal Component Analysis (PCA) and Uniform Manifold Approximation and Projection (UMAP). Unsupervised spatial clustering was enforced using the top 30 principal components under default resolution parameters. Topographical gene expression signatures were directly mapped onto tissue sections utilizing the SpatialFeaturePlot module. Parallel spatial matrix processing was conducted within a Python environment using the Scanpy framework (Wolf et al., 2018). Finally, to precisely delineate and validate the tumor invasive margins across the spatial architecture, topographical coordinates were computationally resolved utilizing the Cottrazm package.

### Immune infiltration, immunotherapy, and pharmacological prediction

Pathway enrichment trajectories were analyzed using the GseaVis algorithm. To quantify functional crosstalk, z-score normalization of the signature genes was performed, and the resulting scores were used to assess correlations between the prognostic model and the relevant biological pathways. The predictive capacity for immune checkpoint blockade (ICB) was independently validated using the IMvigor210 cohort (Necchi et al., 2017) (from a phase II study of atezolizumab treatment). Model-derived algorithms were applied to this dataset to assess clinical immunotherapy responses. Furthermore, transcriptomic profiles of human cancer cell lines (CCLs) were extracted from the Cancer Cell Line Encyclopedia database (Subramanian et al., 2005). Corresponding pharmacological metrics were systematically queried from the CTRP (v2.0) and PRISM Repurposing arrays. Drug response was evaluated based on the area under the curve (AUC). Finally, therapeutic susceptibility was computationally compared across the stratified risk-defined patient subgroups. In parallel, protein–protein interaction (PPI) analysis was performed in NetworkAnalyst 3.0 using experimentally validated data from STRING v11.0. Genes associated with TRIP6 were identified in the TCGA cohort using |R| > 0.2 and *P* < 0.05, followed by KEGG enrichment analysis. A network proximity-based drug repositioning framework integrating DrugBank targets and the STRING interactome was then applied to prioritize candidate compounds, with statistical significance assessed by 10,000 random permutations and multiple-testing correction.

### Cell culture and siRNA transfection

The normal human colonic epithelial lineage (CCD-841) and corresponding malignant lineages (HCT116 and SW480) were sourced from the American Type Culture Collection (ATCC, United States). Routine cellular maintenance was strictly executed in Dulbecco’s Modified Eagle Medium (DMEM, Gibco, United States). The basal medium was enriched with 10% fetal bovine serum (FBS, Gibco, United States) alongside a 1% penicillin-streptomycin mixture. Standard atmospheric parameters for the incubation were locked at 37 °C with 5% CO_2_. For the precise targeted silencing of the TRIP6 gene, specific small interfering RNAs (siRNAs) and a non-targeting scramble control (si-NC) were custom-synthesized (GenePharma, Shanghai, China) (Table 1). The designated sequence coordinates were defined as follows:

**TABLE 1:** The target sequences for small interfering RNA and overexpression constructs.

Gene	Sense/Forward (5′–3′)
si-TRIP6#1	GCC​UAA​AGC​UGA​UCC​UAA​ATT
si-TRIP6#2	GGA​CCU​CAC​UGU​UGG​AUA​ATT

For the *in vitro* interference model, SW480 populations were distributed into 6-well culture scaffolds. Upon reaching an optimal density threshold of 60%–70% confluence, lipid-mediated transfection was initiated utilizing the Lipofectamine 3000 reagent (Invitrogen, United States). Standard commercial protocols were strictly applied. Subsequent downstream functional evaluations were exclusively conducted 48–72 h post-transfection.

### RNA extraction and quantitative real-time PCR (RT-qPCR)

Total RNA was isolated from cultured cells using TRIzol reagent (Invitrogen, United States). cDNA was generated from 1 µg of total RNA using the PrimeScript RT Reagent Kit (TaKaRa, Japan). Quantitative real-time PCR was carried out with SYBR Green Master Mix (Vazyme, China) on an ABI 7500 Real-Time PCR System (Applied Biosystems, United States). TRIP6 mRNA levels were normalized against GAPDH and determined using the 2^−ΔΔCt^ method.

### Western blot analysis

Cells were lysed in RIPA buffer supplemented with protease and phosphatase inhibitors (Roche, Switzerland). Protein concentration was measured with a BCA kit (Thermo Fisher Scientific). Equal amounts of protein were resolved on 10% SDS-PAGE gels and transferred to PVDF membranes (Millipore, United States). After blocking with 5% non-fat milk, membranes were incubated overnight at 4 °C with antibodies against E-cadherin, N-cadherin, Vimentin, and GAPDH (Cell Signaling Technology, United States ; 1:1000), followed by HRP-linked secondary antibodies (1:5000). Signals were visualized using an ECL system (Bio-Rad, United States), and band density was analyzed with ImageJ.

### Cell migration assays

For the Transwell migration assay, 5 × 10^4 transfected SW480 cells in 200 µL serum-free DMEM were added to the upper chamber (8-µm pore size, Corning, United States), while 600 µL DMEM containing 10% FBS was placed in the lower chamber. After 24 h, non-migrated cells were removed, and migrated cells were fixed, stained with 0.1% crystal violet, and counted in five random fields under an inverted microscope. For the wound-healing assay, transfected cells were grown to confluence in 6-well plates and scratched with a sterile 200 µL pipette tip. After PBS washing, cells were maintained in serum-free medium, and wound images were recorded at 0 and 48 h. Migration was quantified as the percentage of wound closure using ImageJ.

### EdU proliferation assay

Cell proliferation was assessed by the 5-ethynyl-2′-deoxyuridine (EdU) assay (Ribobio, Guangzhou, China). Briefly, transfected SW480 cells were exposed to 50 µM EdU for 2 h at 37 °C. The cells were then collected, fixed, permeabilized, and processed with the Apollo reaction according to the manufacturer’s instructions. The proportion of EdU-positive cells was measured by flow cytometry (BD Biosciences, United States).

### Cell apoptosis assay

Apoptosis was measured using the Annexin V-FITC/PI Apoptosis Detection Kit (BD Biosciences, United States). Transfected cells were collected with EDTA-free trypsin, washed twice with cold PBS, and resuspended in 1× binding buffer. Cells were then stained with 5 µL Annexin V-FITC and 5 µL PI for 15 min at room temperature in the dark. The apoptotic rate was quantified by flow cytometry.

## Results

### Construction of the single-cell transcriptomic landscape

**FIGURE 1:**
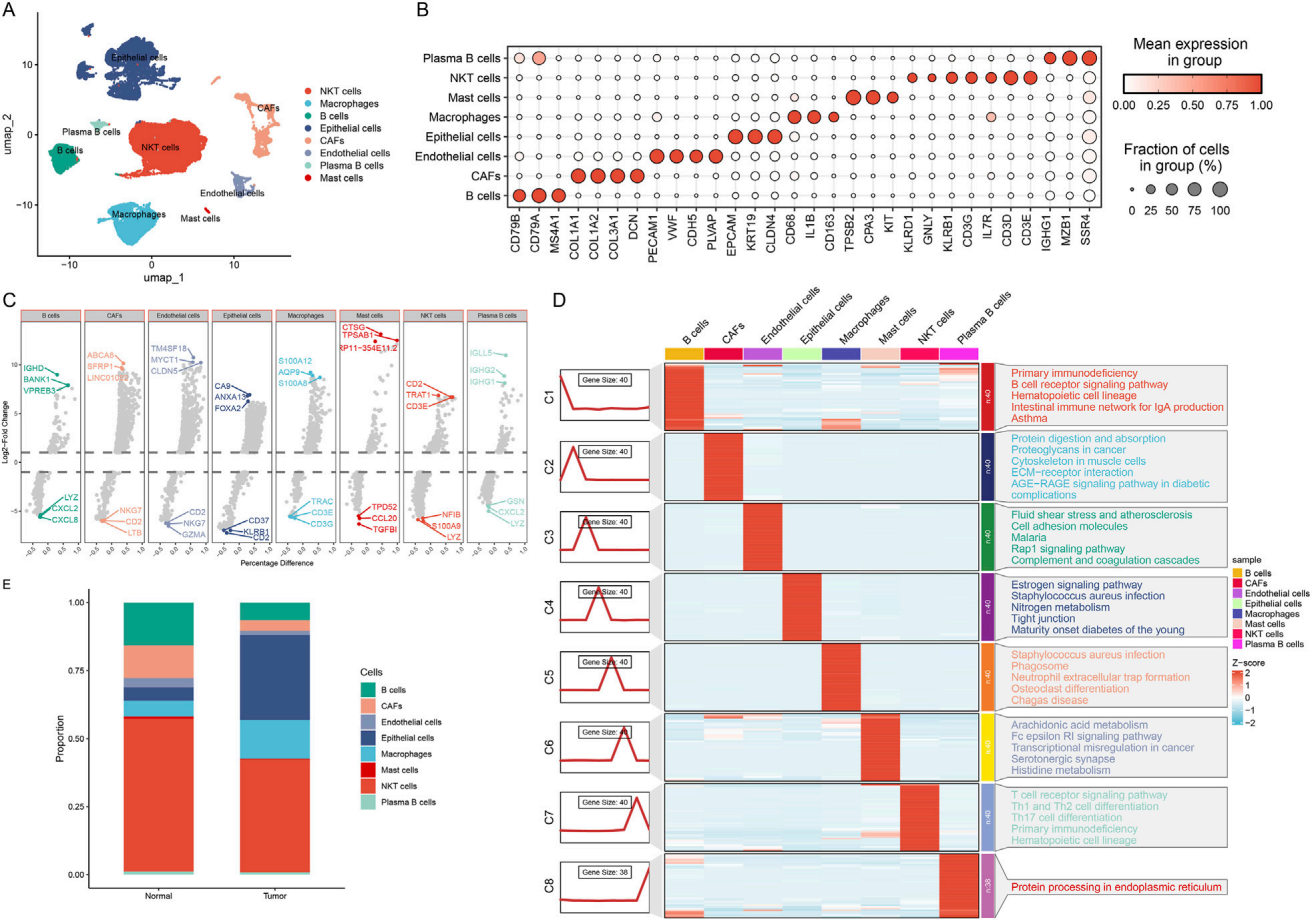
Computational reconstruction of the single-cell transcriptomic landscape. **(A)** Uniform Manifold Approximation and Projection (UMAP) mapping of the annotated cellular subpopulations post-dimensionality reduction. **(B)** Transcriptomic expression dynamics of canonical marker genes across the distinct cellular lineages. **(C)** Volcano plots illustrating the statistical distribution of cluster-specific DEGs. **(D)** KEGG pathway functional mapping derived from the population-specific transcriptomic signatures. **(E)** Comparative analysis of cellular fractional compositions between the malignant and adjacent normal microenvironments.

Following stringent quality filtering and dimensionality reduction, the cellular landscape was resolved into 21 distinct unsupervised clusters. Based on canonical transcriptomic signatures, these clusters were precisely mapped to eight major cellular lineages (Figure 1A). The annotated populations encompassed Epithelial cells, CAF, Macrophages, NKT cells, B cells, Endothelial cells, Mast cells, and Plasma B cells. Cluster-specific differentially expressed genes (DEGs) were subsequently extracted. The high-fidelity expression heatmaps (Figure 1B) and the statistical distribution of log-fold changes (Figure 1C) strongly cross-validated the accuracy of the cell-type assignment. Functional enrichment algorithms applied to these marker genes revealed highly compartmentalized biological roles across different populations (Figure 1D). Specifically, B and NKT lineages demonstrated dense mapping to immune-response cascades, including primary immunodeficiency and B/T-cell receptor signaling. Conversely, CAF populations were strongly linked to matrix remodeling networks, such as protein digestion and ECM-receptor interactions. These computational readouts confirm that the distinct subpopulations execute highly specialized roles within the tumor microenvironment. Furthermore, cellular fraction analysis highlighted a distinct structural transformation between normal and malignant states (Figure 1E). Within the tumor microenvironment, the epithelial fraction exhibited massive expansion, paired with a severe depletion of immune-infiltrating clusters, notably NKT and B cells. This dynamic compositional shift directly highlights the extensive single-cell heterogeneity of colorectal cancer and computationally indicates a fundamentally immunosuppressive microenvironment.

### Computational quantification of cellular stemness and genomic instability

**FIGURE 2:**
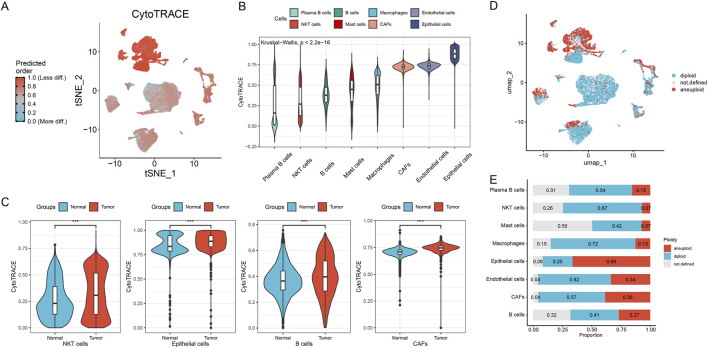
Algorithmic evaluation of cellular stemness and genomic alterations. **(A)** UMAP visualization of CytoTRACE-inferred transcriptomic stemness gradients. Red spectral shifts mathematically denote elevated stemness and reciprocal dedifferentiation states. **(B)** Statistical distribution of the computed stemness indices across the major annotated cellular lineages. **(C)** Comparative quantification of stemness scores across specific cellular compartments, strictly stratified by malignant and adjacent normal microenvironments. **(D)** Topographical UMAP projection of inferred CNVs mapped onto the single-cell architecture. **(E)** Fractional composition of aneuploid versus diploid genomic states systematically quantified across distinct cell populations.

To further evaluate the differentiation potential and stemness features of distinct cell populations within the colorectal cancer microenvironment at single-cell resolution, we applied the CytoTRACE algorithm to quantify cellular stemness states. Dimensionality reduction visualization revealed a markedly heterogeneous distribution of stemness across the overall microenvironment (Figure 2A). Quantitative comparison across cell types further showed significant differences in stemness scores among cell populations. Notably, epithelial cells exhibited the highest stemness scores, significantly exceeding those of all other subsets, followed by endothelial cells and CAFs, whereas immune populations such as plasma cells, NKT cells, and B cells showed generally low stemness levels (Figure 2B). These findings suggest that epithelial cells in colorectal cancer possess strong dedifferentiation capacity and stem-like features, whereas immune cells are predominantly in highly mature and terminally differentiated states. To further explore the potential impact of the tumor microenvironment on cellular differentiation status, we compared stemness differences between normal and tumor samples within the same cell type. The results indicated that tumor development was accompanied by widespread remodeling of cellular stemness. Specifically, epithelial cells in tumor tissues exhibited significantly higher CytoTRACE scores than their counterparts in normal tissues (Figure 2C). Notably, this trend was not limited to epithelial cells; multiple stromal and immune cell populations, also showed significantly elevated stemness scores in tumor samples (Figure 2C; Supplementary Figure S1). These findings imply that the colorectal cancer microenvironment not only endows tumor cells with enhanced stemness and malignant proliferative potential, but may also induce dedifferentiation or phenotypic state changes in resident stromal and immune cells through paracrine mechanisms.

We next used copyKAT to assess CNV in epithelial cells. According to the analysis, cells classified as aneuploid were considered to harbor CNV alterations, whereas diploid cells were regarded as lacking obvious CNV changes. We then examined the proportion of CNV-positive cells at the single-cell level and found that 66% of epithelial cells were classified as aneuploid, whereas the proportion was much lower in immune-related populations such as T cells and B cells (Figures 2D,E). Taken together, these results, combined with the elevated stemness scores, indicate that epithelial cells had undergone malignant transformation.

### Computational mapping of cellular pathways and metabolic networks

**FIGURE 3:**
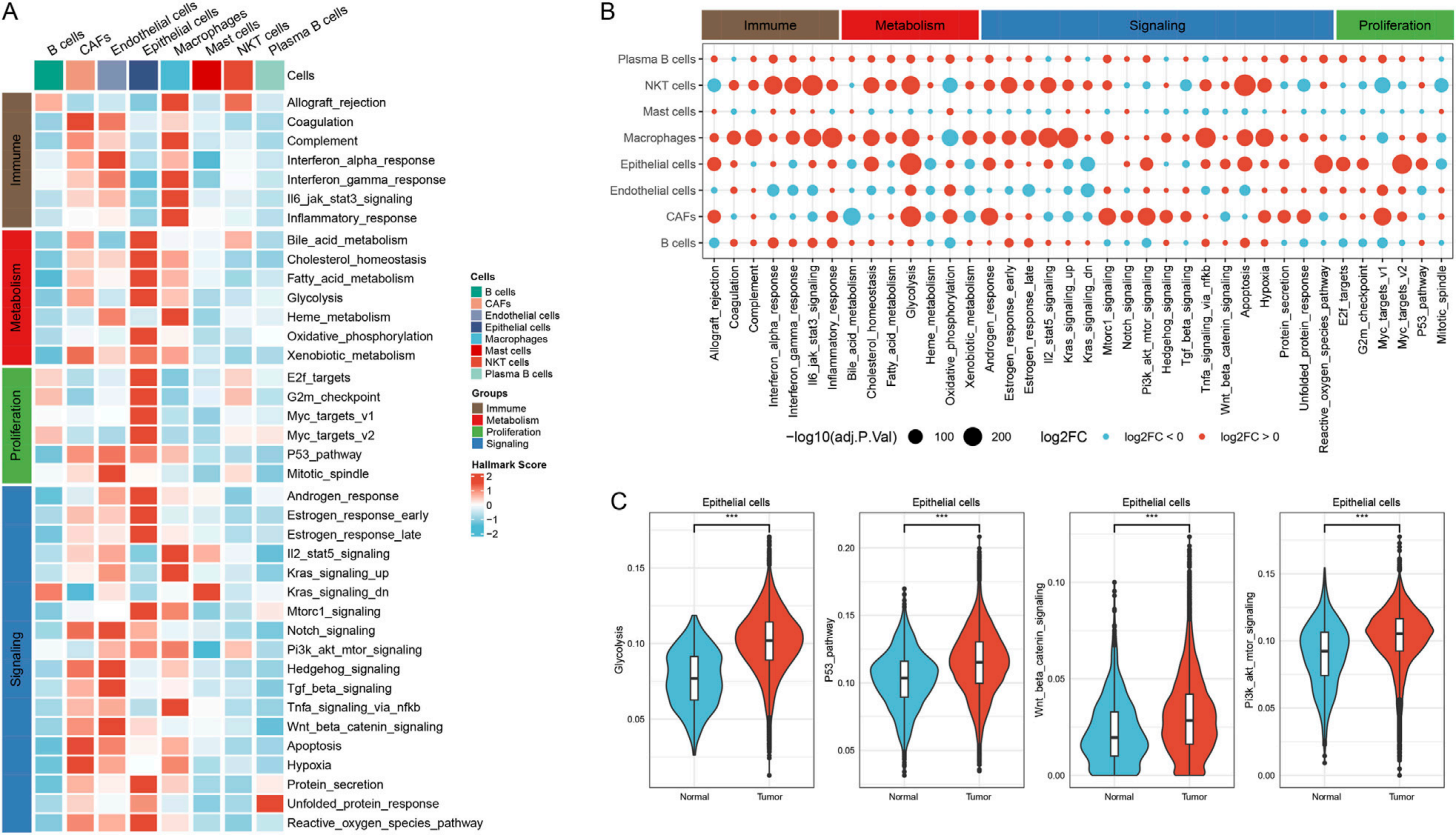
Computational mapping of cellular pathways and metabolic networks. **(A)** Topographical heatmap detailing the differential enrichment gradients of four core functional modules across distinct single-cell lineages. **(B)** Systematic comparative profiling of the four categorical functional metrics. The algorithmic readouts are strictly stratified by cellular identity and spatial origin. **(C)** Quantitative cross-comparison of specific oncogenic and metabolic cascade scores exclusively within the defined epithelial compartment, evaluated across tumor and normal states.

Using the AUCell algorithm, we quantified pathway activity across all cell populations based on the classical Hallmark gene sets and further categorized these pathways into four functional modules: Immune, Metabolism, Signaling, and Proliferation (Figure 3A). Heatmap visualization revealed strikingly distinct pathway activation patterns among different cell populations. Malignant epithelial cells exhibited markedly elevated activity in proliferation- and metabolism-related pathways, whereas their scores for immune-response pathways remained relatively low. In contrast, macrophages and NKT cells displayed strong immune signaling features. To further characterize the dynamic changes in pathway activity across cell subsets during tumorigenesis, we used the limma algorithm to assess differences in pathway scores between tumor and normal samples for each cell type (Figure 3B). The bubble plot demonstrated that the establishment of the tumor microenvironment was accompanied by extensive transcriptional reprogramming, with pathway alterations showing high cell type specificity. Notably, epithelial cells—the principal cells of origin in colorectal cancer—displayed aberrant activation of multiple classical oncogenic and metabolic pathways in tumor tissues. Quantitative comparison showed that, relative to normal tissues, tumor-derived epithelial cells had significantly increased activity scores for Glycolysis, the P53 pathway, Wnt/β-catenin signaling, and the PI3K-AKT-mTOR signaling axis (*P* < 0.001) (Figures 3B,C). These findings not only provide multidimensional evidence for the highly proliferative and metabolically reprogrammed state of malignant epithelial cells, including Warburg-like metabolic features, but also closely align with canonical molecular mechanisms implicated in colorectal cancer progression, such as aberrations in the Wnt and PI3K pathways.

### Algorithmic decoding of intercellular communication networks

**FIGURE 4:**
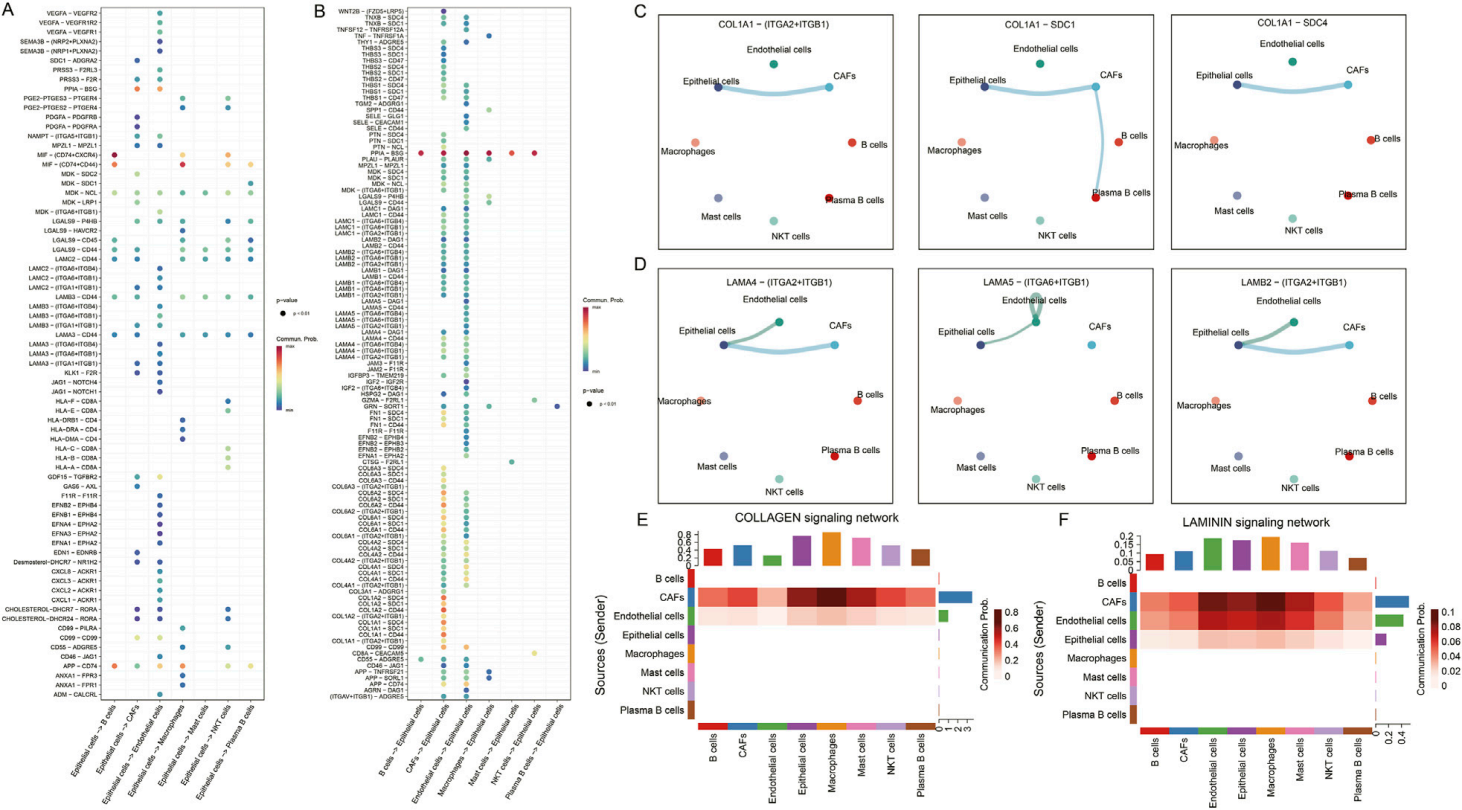
CellChat-based analysis of tumor microenvironment cell–cell communication networks and ligand–receptor interactions. **(A,B)** Bubble plots showing significantly enriched ligand–receptor interactions between epithelial cells and other microenvironmental cell subsets when epithelial cells act as the signal sender **(A)** or signal receiver **(B)**, respectively. **(C,D)** Interaction network plots illustrating the specific receptor–ligand binding patterns and target relationships in the COLLAGEN and LAMININ signaling pathways. **(E,F)** Heatmaps showing the distribution and intensity of communication probabilities among different cell subsets as signal sources/senders and receivers within the COLLAGEN and LAMININ signaling networks.

To characterize intercellular communication in the colorectal cancer microenvironment, we applied CellChat to infer ligand–receptor interaction networks. Bubble plot analysis indicated that malignant epithelial cells acted not only as active signal senders, with VEGFA- and MDK-related signaling potentially linked to angiogenesis and microenvironmental remodeling (Figure 4A), but also as major signal receivers engaged in extensive bidirectional communication with cancer-associated fibroblasts (CAFs) (Figure 4B). Given this prominent interaction pattern, we further focused on ECM-related signaling pathways. Heatmap analysis showed that CAFs and endothelial cells were the dominant senders of COLLAGEN and LAMININ signals, whereas epithelial cells were the principal receiving population (Figures 4E,F). At the molecular level, CellChat predicted that CAF-derived COL1A1 and endothelial/CAF-derived LAMA4/5 may interact with epithelial receptors, including integrin complexes (e.g., ITGA2+ITGB1 and ITGA6+ITGB1) and syndecan receptors (SDC1/4) (Figures 4C,D). Together, these results suggest the presence of active ECM-related stromal–epithelial communication patterns in colorectal cancer. Such predicted interactions are consistent with a microenvironmental context that may support EMT-associated phenotypic plasticity and invasive behavior, although these computational results do not on their own demonstrate direct regulation of TRIP6 or EMT.

### Algorithmic construction and multi-cohort validation of the prognostic risk architecture

**FIGURE 5:**
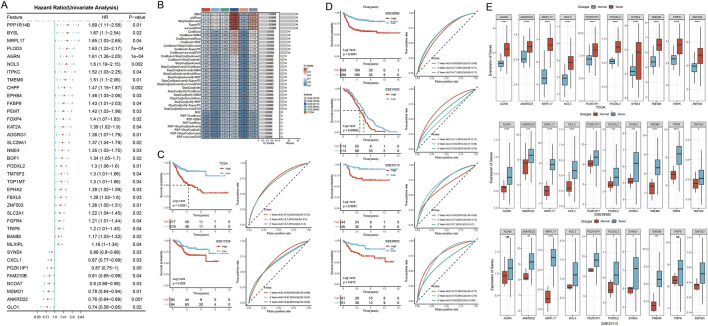
Construction of the prognostic model. **(A)** Univariate Cox regression results in the TCGA cohort. **(B,C)**-index values of 36 machine-learning algorithm combinations across datasets. **(C,D)** Kaplan–Meier survival curves and time-dependent ROC analyses of the prognostic model. **(E)** Expression differences of model genes between normal and tumor tissues in the TCGA-COAD, GSE39582, and GSE33113 datasets.

Univariate Cox regression of epithelial marker genes in the TCGA cohort identified 37 genes associated with overall survival (OS) (Figure 5A). These candidates were then incorporated into a machine-learning screening framework using multiple algorithm combinations. Comparison of Harrell’s C-index across all cohorts indicated that the CoxBoost + StepCox (backward) model provided the best overall prognostic performance (Figure 5B). Patients were therefore classified into high- and low-risk groups according to the median risk score. Kaplan–Meier analysis showed significantly worse OS in the high-risk group in the TCGA training set and all five GEO validation cohorts (*P* < 0.05). Time-dependent ROC curves further supported the predictive value of the model for 1-, 3-, and 5-year survival across datasets (Figures 5C,D). We next assessed the expression patterns of the 10 genes included in the final model. In three bulk transcriptomic cohorts, most genes were differentially expressed between tumor and normal tissues (Figure 5E).

### Clinical translation of the prognostic architecture and isolation of the core therapeutic target

**FIGURE 6:**
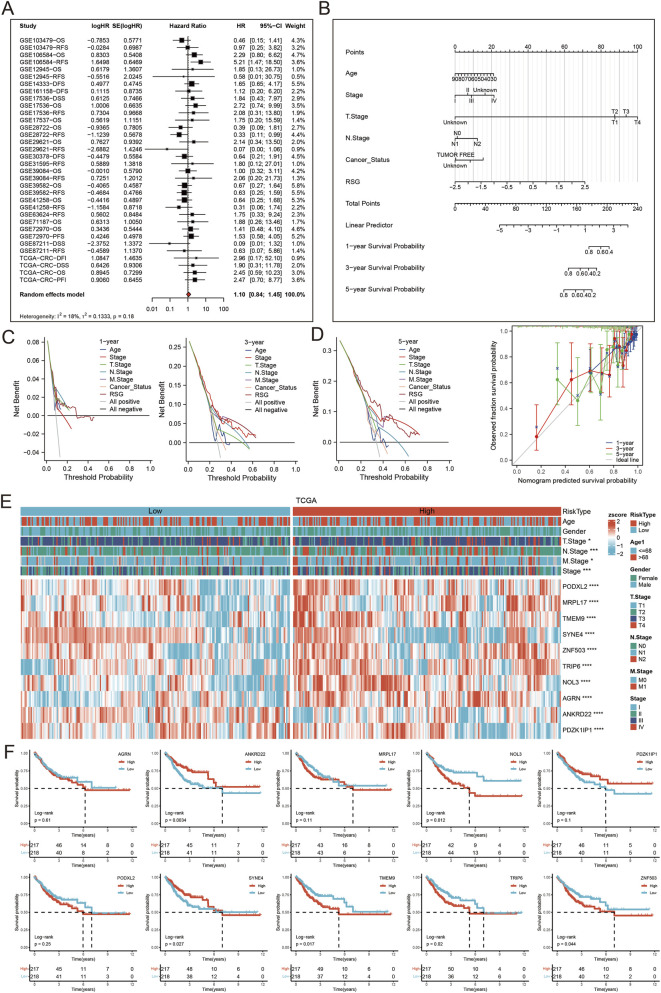
Clinical utility of the prognostic model and survival analysis of individual model genes. **(A)** Forest plot summarizing the meta-analysis of hazard ratios for the risk model in predicting OS, DFS, and RFS across independent TCGA and GEO cohorts. **(B)** Nomogram combining the risk score with clinicopathological variables to estimate 1-, 3-, and 5-year OS in colorectal cancer. **(C)** Decision curve analysis of the nomogram for 1-, 3-, and 5-year survival prediction. **(D)** Calibration plots comparing nomogram-predicted and observed OS at 1, 3, and 5 years. **(E)** Heatmap showing the expression of the 10 model genes in the high- and low-risk groups of the TCGA-COAD cohort, together with clinicopathological features, including age, sex, and TNM stage. **(F)** Kaplan–Meier curves showing the association between expression of the 10 model genes and overall survival. Higher expression of genes such as TRIP6 was associated with worse prognosis.

To assess the robustness and clinical utility of the risk model, we first conducted a meta-analysis across multiple independent colorectal cancer cohorts. As shown in the forest plot, the model showed a consistent prognostic effect in most datasets, with HRs >1. The pooled results further supported the risk score as a reliable predictor of OS and disease-free/recurrence-free survival (DFS/RFS) in colorectal cancer (Figure 6A). To improve clinical applicability, we combined the risk score with age and TNM stage to establish a nomogram for predicting 1-, 3-, and 5-year survival (Figure 6B). Calibration plots showed good agreement between predicted and observed outcomes (Figure 6D). In addition, decision curve analysis (DCA) demonstrated that the combined model provided greater clinical benefit than conventional clinicopathological factors alone across different time points (Figures 6C,D). We further used a heatmap to display the distribution of the 10 model genes and clinicopathological characteristics between the high- and low-risk groups. Patients in the high-risk group tended to have more advanced clinical stage, as well as higher T and N stages (Figure 6E), in line with the adverse prognosis indicated by the model. Finally, to identify potential key targets for subsequent experimental validation, we performed Kaplan–Meier survival analysis for each of the 10 model genes individually (Figure 6F). Several genes showed significant associations between high expression and worse OS. Among them, TRIP6 was of particular interest because it not only showed significant prognostic relevance, but also exhibited preferential overexpression in malignant epithelial cells according to our previous single-cell analysis. Based on these findings, we hypothesized that TRIP6 may function as an important regulator of colorectal cancer progression and therefore selected it as a candidate target for subsequent functional validation.

### Molecular pathway characteristics of the prognostic risk model, potential drug screening, and prediction of immunotherapy response

**FIGURE 7:**
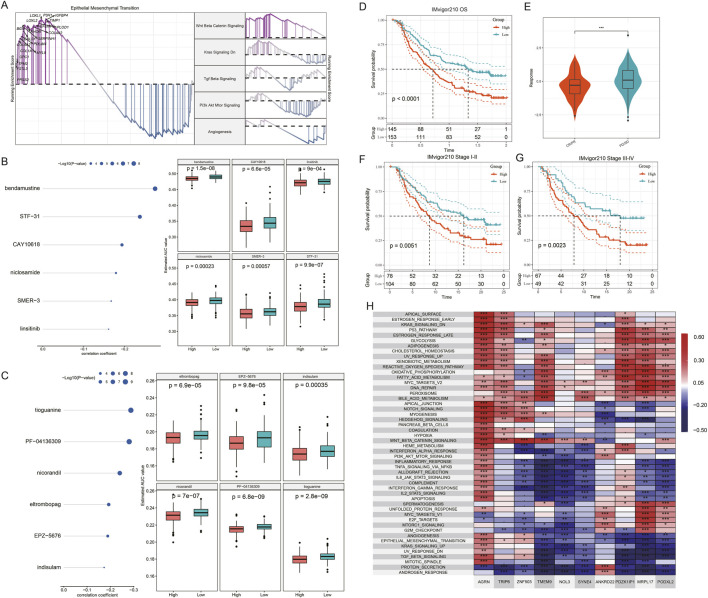
Pathway activation patterns, predicted drug sensitivity, and immunotherapy benefit associated with the prognostic risk model. **(A)** GSEA showing significantly differentially activated biological pathways between the high- and low-risk groups. **(B,C)** Drug sensitivity analyses based on the CTRP and PRISM databases. **(D)** Kaplan–Meier survival curves comparing OS between high- and low-risk patients in the overall IMvigor210 cohort. **(E)** Violin/box plots showing the quantitative difference in prognostic risk scores between immunotherapy responders and non-responders. **(F,G)** Kaplan–Meier survival curves comparing OS between high- and low-risk patients in the stage I–II and stage III–IV subgroups of the IMvigor210 cohort, respectively. **(H)** Heatmap showing the Spearman correlations between the 10 prognostic model genes and the activities of classical Hallmark signaling pathways.

To investigate the molecular basis underlying the poor prognosis of high-risk patients, we performed GSEA. The results showed that the high-risk group was significantly enriched in multiple canonical oncogenic and stromal remodeling pathways, including EMT, Wnt/β-catenin signaling, and TGF-β signaling (Figure 7A). Subsequent Hallmark pathway correlation analysis further confirmed that most model genes, including the core target TRIP6, were strongly co-expressed with these protumorigenic pathways (Figure 7H). Notably, TRIP6 expression was strongly positively correlated with the activation of EMT and angiogenesis pathways, suggesting that TRIP6 may be involved in invasive progression and distant metastasis in colorectal cancer through the promotion of EMT and microenvironmental remodeling. We next evaluated compound sensitivity using the CTRP and PRISM databases, in which a lower AUC indicates higher predicted drug sensitivity. High-risk patients had significantly lower predicted AUC values for bendamustine, CAY10618, tioguanine, and PF-04136309 than low-risk patients (*P* < 0.001, Figures 7B,C). Given the close association of EMT and TGF-β signaling with immune evasion, we next tested the model in the IMvigor210 anti-PD-L1 cohort. High risk scores were associated with worse survival in the overall cohort and stage-defined subgroups (Figures 7D,F,G), and were significantly higher in patients with PD/SD than in those with CR/PR (*P* < 0.001, Figure 7E). These findings support the utility of the model for identifying patients with primary resistance to immune checkpoint therapy.

To further dissect the regulatory mechanism at the level of protein–protein interactions, we constructed a PPI network and identified an interaction module centered on TRIP6 and its associated partners. This core module, together with the 1,616 co-expressed genes, was significantly enriched not only in the ECM-receptor interaction pathway but also in multiple canonical oncogenic cascades, including Wnt, mTOR, and VEGF signaling (Supplementary Figure S2A-B,D). Given the prolonged timeline typically required for *de novo* drug development against a single target, we next introduced a network proximity scoring framework based on systems biology to explore potential drug repurposing strategies targeting the TRIP6-centered module. Specifically, the shortest topological distances between DrugBank-derived drug target sets and the TRIP6 co-expression module were calculated within the interactome, followed by correction through 10,000 random permutation simulations (Supplementary Figure S2C). Using this approach, we ultimately identified 23 candidate compounds with close network proximity to the TRIP6 module, including Archexin, Perifosine, and arsenic trioxide (Supplementary Table S1). These high-confidence candidates may provide a rational therapeutic resource for systemically disrupting the TRIP6-driven malignant signaling axis, and offer potentially valuable references for precision treatment in high-risk patients.

### Spatial transcriptomics reveals the spatial distribution of TRIP6

**FIGURE 8:**
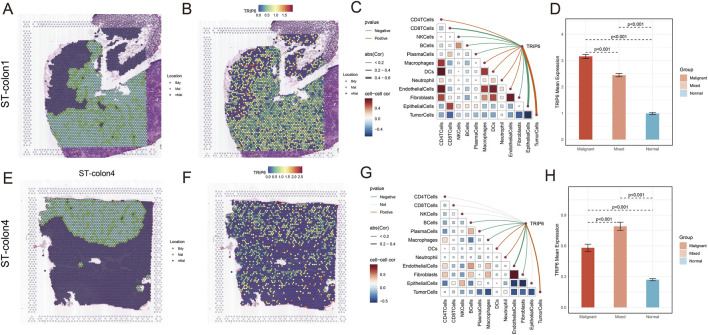
Spatial transcriptomics reveals the spatial distribution of TRIP6. **(A)** Anatomical region annotation of the colorectal cancer spatial transcriptomic section ST-colon1. **(B)** Spatial expression map of TRIP6 in ST-colon1. A deeper red color indicates a higher transcript abundance. **(C)** Correlation network showing the spatial associations between TRIP6 expression and the abundance of different microenvironmental cell types in ST-colon1. **(D)** Bar plot quantitatively comparing the mean expression level of TRIP6 among the malignant, boundary, and normal regions in ST-colon1 (*P* < 0.001). **(E)** Anatomical region annotation of the colorectal cancer spatial transcriptomic section ST-colon4. **(F)** Spatial expression map of TRIP6 in ST-colon4. A deeper red color indicates a higher transcript abundance. **(G)** Correlation network showing the spatial associations between TRIP6 expression and the abundance of different microenvironmental cell types in ST-colon4. **(H)** Bar plot quantitatively comparing the mean expression level of TRIP6 among the malignant, boundary, and normal regions in ST-colon4 (*P* < 0.001).

We included two colorectal cancer spatial transcriptomic samples (ST-colon1 and ST-colon4) and annotated the sections into three regions: malignant (Malignant), tumor–stroma boundary (Mixed), and normal non-malignant (Figures 8A,E). Spatial expression mapping showed that TRIP6 transcripts were enriched in the tumor core and invasive boundary, whereas their expression was relatively low in normal mucosal and stromal regions (Figures 8B,F). Quantitative analysis further confirmed that TRIP6 expression was significantly higher in both the malignant and boundary regions than in the normal region (*P* < 0.001). Notably, in the ST-colon4 sample, TRIP6 expression in the Mixed region was significantly higher than that in the Malignant region (Figures 8D,H). Spatial correlation analysis further showed that higher TRIP6 expression was associated with the local abundance of tumor cells, fibroblasts, endothelial cells, and macrophages (Figures 8C,G), and inversely associated with CD8^+^ T cells, NK cells, and B cells. Together, these results support that TRIP6 exhibits a non-random spatial distribution in colorectal cancer tissues, with preferential enrichment in malignant and boundary regions. The spatial data therefore provide contextual support for the relevance of TRIP6 in aggressive tumor regions, although they do not by themselves establish a direct functional role in invasion or microenvironmental regulation.

### TRIP6 drives the malignant proliferation and migration of colorectal cancer cells

**FIGURE 9:**
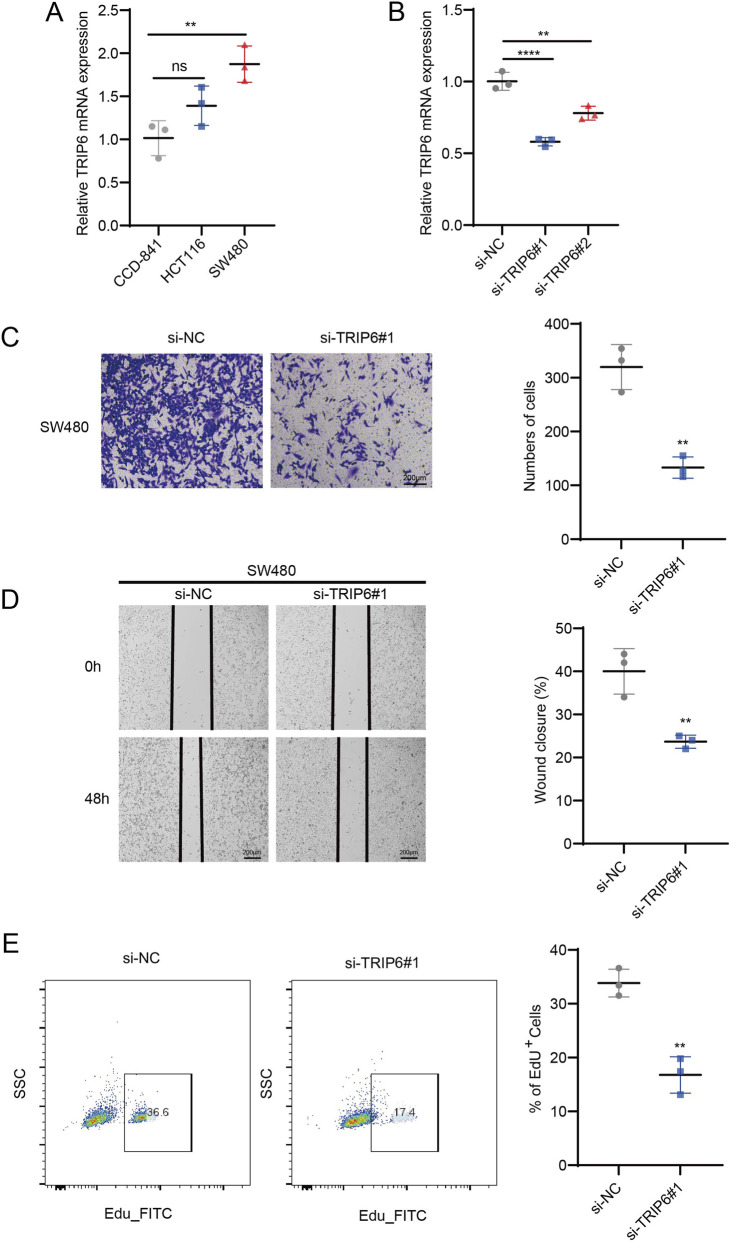
TRIP6 knockdown suppresses colorectal cancer cell proliferation and migration *in vitro*. **(A)** Basal TRIP6 mRNA levels in the normal colonic epithelial cell line CCD-841 and colorectal cancer cell lines HCT116 and SW480, as determined by RT-qPCR. **(B)** RT-qPCR validation of the knockdown efficiency of si-TRIP6#1 and si-TRIP6#2 in SW480 cells. **(C)** Transwell assay showing the effect of TRIP6 silencing on SW480 cell migration, with quantification of migrated cells shown on the right. **(D)** Wound-healing assay evaluating migratory changes in SW480 cells after TRIP6 knockdown, with quantification of wound closure shown on the right. **(E)** EdU flow cytometric analysis of proliferative activity in SW480 cells following TRIP6 silencing, with quantification of EdU-positive cells shown on the right. Data are presented as mean ± SD. ns, not significant; **P < 0.01; ****P < 0.0001.

Further validation focused on TRIP6. RT-qPCR showed that TRIP6 was markedly upregulated in colorectal cancer cell lines (HCT116 and SW480) relative to the normal colonic epithelial cell line CCD-841 (Figure 9A, *P* < 0.05), consistent with the spatial transcriptomic findings. Because SW480 cells showed relatively high endogenous TRIP6 expression, they were selected for siRNA-mediated knockdown. Among the tested siRNAs, si-TRIP6#1 achieved the highest knockdown efficiency and was used in subsequent experiments (Figure 9B, *P* < 0.01). Given that spatial transcriptomics indicated preferential TRIP6 expression at the tumor invasive front, we next examined its role in cell motility. Transwell and wound-healing assays showed that TRIP6 silencing significantly impaired migration and delayed wound closure at 48 h compared with the si-NC group (Figures 9C,D, *P* < 0.01). Flow cytometry further showed that TRIP6 knockdown reduced the fraction of EdU-positive cells from 33.6% to 17.4% (Figure 9E, *P* < 0.01), indicating a marked suppression of proliferative activity.

**FIGURE 10:**
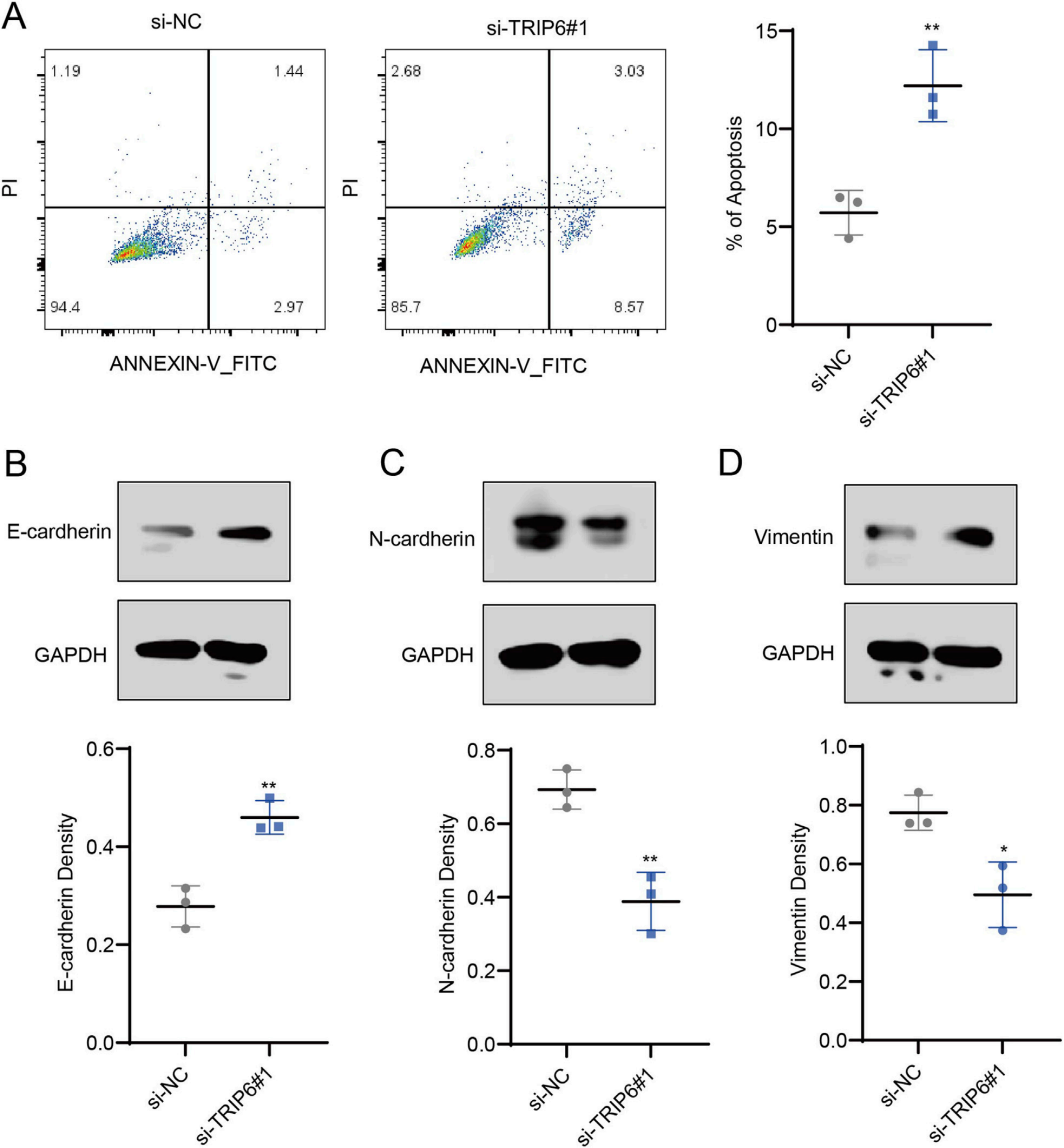
TRIP6 knockdown induces apoptosis and suppresses the EMT process in colorectal cancer cells. **(A)** Apoptosis of SW480 cells transfected with si-NC or si-TRIP6#1 was assessed by Annexin V-FITC/PI double staining followed by flow cytometry. Representative flow cytometric plots are shown on the left, and the numbers in the right quadrants indicate the combined proportion of early and late apoptotic cells. Quantitative analysis of the total apoptotic rate (% apoptosis) is presented on the right. **(B-D)** Western blot analysis was performed to evaluate the effects of TRIP6 knockdown on the protein expression levels of EMT-related markers.*P < 0.05; **P < 0.01.

Subsequently, apoptosis was assessed by flow cytometry. The results showed that, compared with cells transfected with the si-NC, si-TRIP6#1 markedly induced both early and late apoptosis in SW480 cells, leading to a significant increase in the total apoptotic rate (Figure 10A, *P* < 0.01). We next examined the expression changes of EMT-related markers. Following TRIP6 knockdown, E-cadherin expression was significantly increased (Figure 10B, *P* < 0.01), whereas N-cadherin and Vimentin were markedly decreased (Figures 10C,D, *P* < 0.05). These findings suggest that TRIP6 contributes to the malignant phenotype of colorectal cancer cells and may be involved in EMT-related regulation. TRIP6 knockdown induced apoptosis and was accompanied by a shift toward a less mesenchymal-like marker profile in colorectal cancer cells.

## Discussion

Colorectal cancer progression is a dynamic process characterized by marked cellular heterogeneity, extensive microenvironmental remodeling, and the progressive accumulation of molecular abnormalities at multiple levels (Bell et al., 2023; Khaliq et al., 2022; Wu et al., 2026a; Liu et al., 2025). Recent advances in single-cell and spatial transcriptomic technologies have provided new opportunities to dissect cellular state transitions and spatial organization within the tumor microenvironment. In the present study, by integrating single-cell RNA sequencing, spatial transcriptomics, multi-cohort bulk transcriptomic data, and machine-learning analysis, we established a prognostic model based on epithelial marker genes and further identified TRIP6 as a candidate gene associated with invasive behavior. Combined with *in vitro* experiments, our findings suggest that TRIP6 may participate in the regulation of CRC cell proliferation, migration, and EMT. Together, these results provide additional multi-omic evidence for understanding the malignant progression of CRC.

First, our single-cell analyses indicate that malignant epithelial cells in CRC exhibit prominent stemness-associated and genomic abnormality-related features. CytoTRACE analysis showed that epithelial cells had significantly higher stemness scores than most other cell populations, while copyKAT analysis further suggested a high proportion of aneuploid alterations in this compartment. These observations are largely consistent with previous evidence showing that tumor evolution is accompanied by dedifferentiation and chromosomal instability. Increased stemness has been closely linked to therapeutic resistance, metastatic potential, and tumor recurrence, whereas copy number alterations often reflect clonal expansion and evolutionary advantage under selective pressure (Tape, 2024; Wang et al., 2021). Our results further support this concept at single-cell resolution, suggesting that CRC epithelial cells acquire enhanced plasticity and malignant potential within the tumor ecosystem.

At the functional level, AUCell analysis showed that malignant epithelial cells were characterized by active metabolic and proliferative programs, whereas immune-response-related pathways remained relatively inactive. Further differential analysis revealed significant enrichment of Glycolysis, Wnt/β-catenin, PI3K-AKT-mTOR, and P53 signaling in tumor-derived epithelial cells. These findings are broadly consistent with the established molecular events in CRC. Aberrant Wnt/β-catenin signaling is widely recognized as a pivotal early event in colorectal tumorigenesis (Wang et al., 2021; Chen et al., 2024), whereas activation of PI3K-AKT-mTOR and metabolic pathways has been implicated in sustained tumor growth, stress adaptation, and invasive phenotypes (Leiphrakpam and Are, 2024). Notably, enhanced glycolysis not only reflects metabolic rewiring in cancer cells but may also reshape the local metabolic milieu and thereby influence immune cell function (Shen et al., 2020; Fang et al., 2019). Although causal relationships among these pathways were not directly examined in this study, our findings suggest that metabolic reprogramming and oncogenic signaling activation may jointly contribute to maintaining the malignant state of CRC epithelial cells.

In addition to cell-intrinsic alterations, intercellular communication within the tumor microenvironment may also play an important role in CRC progression. CellChat analysis revealed substantial bidirectional communication between malignant epithelial cells and CAFs, particularly in ECM-related signaling pathways such as COLLAGEN and LAMININ, in which CAFs and endothelial cells acted as major signal senders and epithelial cells as principal receivers (Luo et al., 2021; Qin et al., 2020). Previous studies have shown that CAF-mediated matrix remodeling, integrin signaling, and changes in ECM stiffness are important extrinsic regulators of EMT and invasion. Our findings are consistent with this view and suggest that CAFs may continuously modulate epithelial cell states through ECM–receptor interactions, thereby contributing to EMT-associated phenotypic remodeling in CRC. However, it should be noted that these results are primarily based on computational inference and do not directly establish a functional requirement for any specific ligand–receptor axis in EMT induction. Further validation using co-culture systems and blockade experiments will be required.

From a translational perspective, we developed a prognostic risk model based on epithelial marker genes and validated it across TCGA and multiple independent GEO cohorts. The model consistently stratified patients into high- and low-risk groups across datasets, suggesting that single-cell-derived epithelial features may carry prognostic value. Compared with conventional prognostic signatures developed solely from bulk transcriptomic differential expression analyses, our model may offer improved biological interpretability by incorporating cell-of-origin information. In addition, the nomogram and decision curve analyses suggested that this model may provide incremental value in survival estimation when combined with clinical variables. Nevertheless, model performance may still be influenced by sample source, platform heterogeneity, and the retrospective nature of the available datasets. Therefore, its clinical utility will require further validation in larger, multicenter, and prospective cohorts. Among the genes included in the model, TRIP6 emerged as a gene of particular interes (Gou et al., 2023; Li et al., 2025). TRIP6 is a LIM domain-containing member of the zyxin family and has been implicated in focal adhesion assembly, cytoskeletal remodeling, signal transduction, and cell migration. Previous studies have linked TRIP6 to proliferation, invasion, or poor prognosis in several tumor types, including breast, gastric, and ovarian cancers (Yang et al., 2020; Daniel et al., 2023). In addition, TRIP6 has been reported to interact functionally with signaling pathways such as LPA receptor signaling, NF-κB and Wnt/β-catenin, thereby contributing to cell motility and epithelial–mesenchymal-associated transcriptional programs (Gou et al., 2019; Li et al., 2005; Li et al., 2026). However, studies on TRIP6 in CRC remain limited, particularly with respect to its spatial localization within the tumor microenvironment and its relationship with local cellular niches.

In the present study, spatial transcriptomic analysis showed that TRIP6 was highly expressed in both the tumor core and the tumor–stroma boundary region. Its expression was positively correlated with the abundance of fibroblasts, endothelial cells, and macrophages, but negatively correlated with certain anti-tumor immune cell populations. This distribution pattern suggests that TRIP6 may be associated with microenvironmental remodeling at the invasive front. Together with our single-cell findings of enhanced ECM communication between CAFs and epithelial cells, these data raise the possibility that TRIP6 may participate in tumor cell responses to microenvironmental cues and in the maintenance of invasive phenotypes. However, these associations are currently based on spatial colocalization and expression correlation, and do not by themselves establish a causal mediating role for TRIP6.

Our *in vitro* experiments further support a possible role for TRIP6 in regulating malignant phenotypes. We found that TRIP6 was upregulated in CRC cell lines compared with normal colonic epithelial cells. Silencing TRIP6 in SW480 cells reduced migratory capacity and proliferative activity, increased apoptosis, and was accompanied by upregulation of E-cadherin and downregulation of N-cadherin and Vimentin. These observations suggest that TRIP6 may contribute to the maintenance of EMT-related phenotypes and growth advantage in CRC cells. However, our current data primarily support an association between TRIP6 and malignant progression-related phenotypes, together with an inhibitory effect of TRIP6 knockdown *in vitro*, rather than definitively establishing TRIP6 as a contributor to CRC invasion and progression. In combination with the enrichment of EMT-, TGF-β-, and Wnt/β-catenin-related pathways in the high-risk group, TRIP6 may represent an important node within a pro-invasive signaling network. Still, the direct downstream mechanisms of TRIP6 remain unresolved, and it is not yet clear whether its effects are mediated through a specific integrin/cytoskeletal axis. Therefore, its mechanistic role should be interpreted with caution.

In addition, we identified several potential candidate drugs using a network proximity-based strategy, providing preliminary clues for therapeutic exploration in high-risk patients. It should be emphasized, however, that network-based drug prediction is primarily hypothesis-generating. The actual efficacy and clinical relevance of these compounds will require further validation in cellular, animal, and clinical settings. Accordingly, these findings should be viewed as a basis for future investigation rather than direct therapeutic recommendations.

Several limitations of this study should also be acknowledged. First, although we integrated multiple public cohorts and performed cross-validation, the overall analysis was still based largely on retrospective datasets, and technical differences across platforms may affect model stability. Second, the number of spatial transcriptomic samples was limited and may not fully capture spatial heterogeneity across CRCs of different stages, molecular subtypes, or metastatic states. Third, functional validation of TRIP6 was restricted to *in vitro* experiments, without support from animal models or clinical tissue-based validation.

## Conclusion

In summary, this study delineates the malignant-state characteristics of epithelial cells in the CRC microenvironment from single-cell, spatial, and bulk transcriptomic perspectives, and establishes a prognostic model with reasonable robustness. We further propose and preliminarily validate TRIP6 as a potential molecule linking microenvironmental signaling to tumor invasive behavior. These findings provide additional insight into microenvironmental remodeling, EMT regulation, and prognostic stratification in CRC, and may offer a basis for future mechanistic and therapeutic studies centered on TRIP6.

## Data Availability

The datasets presented in this study can be found in online repositories. The names of the repository/repositories and accession number(s) can be found below: https://www.ncbi.nlm.nih.gov/geo/, GSE161277, GSE132465 https://www.ncbi.nlm.nih.gov/geo/, GSE17538, GSE33113, GSE39582, GSE14333, GSE38832 https://portal.gdc.cancer.gov/, TCGA-COAD.

## Supplementary material

SUPPLEMENTARY FIGURE S1 Comparison of CytoTRACE-inferred stemness scores between normal and tumor tissues across major cell populations in the colorectal cancer microenvironment. Violin plots with embedded boxplots show the distribution of CytoTRACE scores in normal and tumor samples across major annotated cell types, including NKT cells, epithelial cells, B cells, CAFs, macrophages, mast cells, endothelial cells, and plasma B cells. Higher CytoTRACE scores indicate lower differentiation status and greater stem-like potential. Compared with their counterparts in normal tissues, multiple cell populations in tumor samples displayed elevated stemness scores, particularly epithelial cells, CAFs, NKT cells, and B cells, suggesting widespread remodeling of cellular differentiation states during colorectal cancer progression. These results support the notion that the tumor microenvironment not only enhances stemness-associated features in malignant epithelial cells but may also induce phenotypic reprogramming in stromal and immune populations. Statistical significance was assessed using the Wilcoxon rank-sum test.

SUPPLEMENTARY FIGURE S2
**(A)** PPI network analysis of MRPL17, TRIP6, AGRN, and NOL3. **(B)** KEGG functional enrichment analysis of genes in the PPI network. **(C)** Drug analysis of TRIP6-related genes. **(D)** KEGG functional enrichment analysis of TRIP6-related genes.
